# A home visit program versus a non-home visit program in total knee replacement patients: a randomized controlled trial

**DOI:** 10.1186/s13018-019-1412-6

**Published:** 2019-11-29

**Authors:** Bura Sindhupakorn, Piya-on Numpaisal, Suwittaya Thienpratharn, Darawan Jomkoh

**Affiliations:** School of orthopedic, Institute of medicine, Suranaree University of Technology, 111 University Ave, Muang District, Nakhon Ratchasima, Nakhon Ratchasima 30000 Thailand

**Keywords:** Home visit program, Non-home visit program, Total knee replacement

## Abstract

**Background:**

The goals in total knee replacement (TKR) are pain relief, restore functions, and improve quality of life. Surgical outcomes were not related to patients’ satisfaction. Low 1-year WOMAC especially in the first 6 weeks and painful TKR related to patient dissatisfied. To improve satisfaction, we created the home visit program (TKR-H) after hospital discharge. INHOMESSS was the rationale for home visit activities.

**Methods:**

We recruited 52 TKRs. Four TKRs were excluded. We used simple randomization for 24 patients as a home visit (TKR-H) and 24 patients as a non-home visit (TKR). Patients were evaluated by general demographics, pain intensity scores (VAS), range of motion (ROM), WOMAC, knee scores, and functional scores as a primary objective. A duration for gait aid independent and patient’s satisfaction score as secondary objective. The study was 6 weeks after surgery.

**Results:**

TKR-H and TKR had significant differences in the mean of WOMAC score (88.29 ± 10.66 vs. 68.00 ± 12.47, respectively, *P* <  0.001), pain score (VAS) (6.25 ± 10.13 vs. 35.67 ± 22.05, respectively, *P* <  0.001), knee score (81.67 ± 10.08 vs. 68.38 ± 6.45, respectively, *P* <  0.001), functional score (77.83 ± 4.22 vs. 73.70 ± 7.48, respectively, *P* = 0.037), and range of motion (107.71 ± 8.47 vs. 98.17 ± 9.57, respectively, *P* = 0.001). The patient’s satisfaction score in TKR-H group (4.71 ± 0.46) was significantly higher than the TKR group (4.13 ± 0.45) (*P* <  0.001) and time to gait aid independent (2.75 ± 0.99 vs. 3.71 ± 1.23, respectively, *P* = 0.005).

**Conclusion:**

Our TKR-H showed better clinical outcomes and satisfaction than non-home visit. The rationale in TKR-H improves satisfaction after total knee replacement.

**Trial registration:**

TCTR20190514001.

## Background

At present, the keys to success for a total knee replacement (TKR) include reducing pain, restoring function, and improving the quality of life [1–3]. Less pain with a wide range of motion and independence are important goals for rehabilitation [4, 5]. Due to the increase in knee replacements worldwide, there is an increasing focus on improving the cost and effectiveness of this procedure with healthcare systems. There is a strong economic pressure to reduce the length of the hospital stay making it the highest priority [6, 7]. However, some researchers have emphasized the risks with early hospital discharge of patients and its impact on their families [8]. The mean 1-year WOMAC score is lowest in the first 3 months [9]. The range of the mean 1 year WOMAC score was 68–82. The Western Ontario and McMaster Universities Arthritis Index (WOMAC) is widely used in the evaluation of Hip and Knee Osteoarthritis. It is a self-administered questionnaire asked about pain, stiffness, and physical function. Numerous studies indicate that only 82% to 89% of patients were satisfied with their TKR. R. B. Bourne [10] found that the factors related to patient satisfaction include pain relief, function for daily living, meeting operative expectations, a low 1-year WOMAC, preoperative pain at rest, and readmission due to complications. One review article [11] showed that the surgical outcome was affected by age, gender, patient’s personality, patient’s expectations, physical and psychological comorbidities, diagnosis for TKR, and the severity of arthropathy. Several authors [12, 13] have compared rehabilitation programs in the hospital with those at home after a TKR and have found no differences in the functional outcomes. But we found no studies that focus on home visits after TKR, especially with the surgeon as the team leader of the home visit team.

Performing home visits is a service model and is part of a health care system that can monitor and assess patients at home. The objectives for home visits are:

1. To provide patients and families with confidence and self-reliance in providing health care at home.
2. To learn about how the family lives and takes care of the patient at home.
3. To completely assess the impact of both disease and illness on the patient and the family.
4. To continue a good long-term relationship between the health care team and the patients and their families.

In order to improve satisfaction, especially in the first 6 weeks, we used a home visit program for patients who had a total knee replacement and patient report outcome measurements (PROMs) for evaluation. Our hypothesis was that home visits would improve the satisfaction after total knee replacement (TKR).

## Materials and methods

**Fig. 1:**
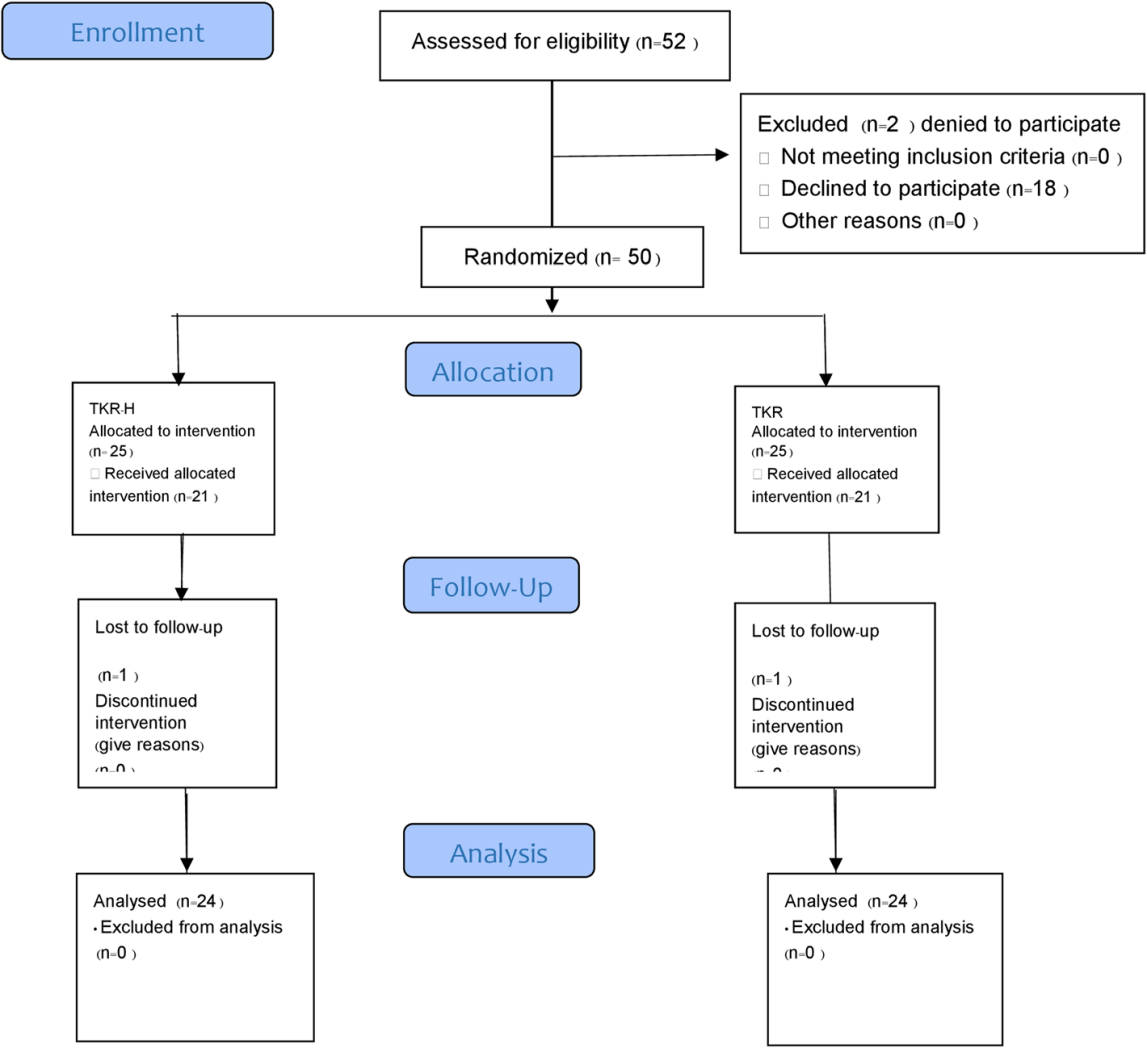
CONSORT 2010 flow diagram

We designed a Clinical Trial study (see Fig. 1). Criteria for selection included that the patients lived within 20 km of Suranaree University of Technology Hospital and had a TKR. We found 52 patients who met the criteria and for the study. Patients signed a patient consent form. Two patients declined to participate. The patients were admitted to the hospital for 3 days. Before discharge, we used simple randomization with 25 patients to receive a home visit and 25 patients for a non-home visit. For both groups, the duration of the study was for the 6 weeks following the TKR and included two follow ups within the 6-week period. The first follow up took place 2 weeks after discharge. For the non-home visits, the meeting place was at an outpatient clinic. For the home visits, we used home visit protocol and the location of the follow up was the patient’s home. The second follow up took place 6 weeks after TKR for a non-home visit and a home visit at the outpatient clinic. We did a satisfaction questionnaire at 6 weeks. We missed a follow up for one patient in each group. Both groups received the same post-operative pain control and rehabilitation protocol. To reduce the confounding factors such as surgical technique, the surgical skill of the surgeon, and type of implants by one experienced surgeon, we used the same medial parapatella technique and the same type fix posterior sacrificed total knee prosthesis for all patients. All complications were collected from both groups. All patients were under the ethics approval and consent from the ethics committee for researchers involving Human subjects, Suranaree University of Technology (EC 60-44), and the patients signed a patient consent form.

### Data collection

Patients were assessed for general demographics and Patient Report Outcome Measurements (PROMs) that included the pain intensity scores using a visual analog scale (VAS) from 0 to 100 mm, knee joint range of motion (ROM), WOMAC, knee scores, function scores, time of independent walking (amount of time before the patients could move independently), and a satisfaction questionnaire for five questions with scores ranging from 1 to 5. The scores indicated the level of satisfaction: score of 5 was very satisfied; score of 4 was satisfied; score of 3 was okay; score of 2 was unsatisfied; and score of 1 was very unsatisfied. All the data from PROMs were recorded by research nurse and at the 6 week after operation.

### Intervention

The participants were assigned to home visit protocol by the researchers prepared the team before the home visit. First, we set of plans and objectives for the visit. Second, we studied the economic and social structures of the community and collected the patient’s medical and family history documents (family folder). Lastly, we did personal preparation such as knowledge, medications, needed equipment, and first aid. During home visits, in order to build trust and relationships for holistic service, the team leader (surgeon) assessed each patient and their family by using the guideline INHOMESSS [14–16]. The following terms were defined: I = immobility. Evaluates whether the patient can take care of themselves or do they need the help of others. We evaluated the patient’s functional activities including an assessment of daily living activities (bathing, transfer, dressing, using the toilet, eating, continence) and instrumental activities of daily life (using the telephone, administering medications, paying bills, shopping for food, preparing meals, doing housework). We asked the patient to demonstrate elements of their daily routine, such as getting out of bed, performing personal hygiene and leisure activities, and getting in and out of a car. Corrective interventions were directed for any noted deficiencies. We assessed the activities of daily living such as using the shower or toilet, dressing, and doing other instrumental activities of daily living. N = nutrition of the patient. Food affects health directly especially for the elderly. We assessed the patient’s current state of nutrition, eating behaviors and food preferences. We started by always asking open-ended questions. For example, “We have been working hard on your diet to control your diabetes. Would you remember the types of foods you eat?” Improvements in cooking materials allowed the physician to assess serving sizes and the nutritional value of foods with relative ease. We also asked questions like, “How many meals do you have per day?” H = home environment. The environmental factors that affect the patients and their families, such as the presence of stairs in the house, whether the bedroom was located upstairs or downstairs, and whether the toilet seat was high or low. The patient’s home environment should allow for privacy, social interaction, and both spiritual and emotional comfort and safety. Important for many older patients is the presence of a safe neighborhood with close proximity to services. The home may reflect the living condition of patients and their families, such as the presence of stairs in the house, the location of the bedroom upstairs or downstairs. O = other people. The relationships within the family. Whether the patient lives alone or with relatives. Also the presence of neighbors who help the patient. By having the patient’s social support system during the home visit, we clarified the roles and concerns of family members. We assessed the availability of emergency help for the patients from family members and their friends and clarified specific issues, such as who is to serve as a surrogate for the patient in the event of incapacitation. Discussion of a living will be more comfortably performed during a home visit than during a clinic visit. Also critically important is the evaluation of the caregiver’s needs and their risk for burnout. M = medications. This factor deals with the history of how the patient takes their medication, their self-reliance and their discipline. To remedy or avoid polypharmacy, we evaluated the type, amount and frequency of medications, and the organization and methods of medication delivery. An inventory of the patient’s medicine containers can provide clues to previously unidentified drug-drug or drug-food interactions. A home medication review can also allow a direct estimate of patient compliance. E = examination. This factor includes physical examination such as blood pressure measurement, wound care, and signs of complications. We did a directed physical examination based on the needs of the patient and the physician’s agenda. We asked the patient to demonstrate proper techniques for walking with or without gait aid. In addition, we can weigh the patient and obtain a blood pressure measurement. In-person correlation of home and office measurements provides useful information aiding in future telephone and clinic contacts. S = spiritual. Health beliefs, attitudes, values, culture, traditions, and psychosocial factors of the patient and their family. We asked about the influence of spiritual beliefs on the patient’s sense of physical and emotional health. This information may provide the impetus, as desired by the patient, for a discussion of spirituality as a coping and healing strategy. S = service. Evaluation at home for available health services for the patient and their family. S = safety. Safety assessment of the home atmosphere. The goal of the home safety assessment is to determine whether the patient’s environment is comfortable and safe (no unreasonable risk of injury). We identified and helped modify potential safety hazards. After each home visit, we collected all data by using the home visit form. This can be used to track data for an improvement of the quality of service. In our study, we used the same discharge preparation protocol for both groups including wound care education, wound management, home medication, and home-based rehabilitation such as range of motion exercises, using a gait aid, and quadriceps exercises.

### Statistical analysis

General demographics including sex, age, pre-op pain score, pre-op range of motion (ROM), and body mass index (BMI). The primary measuring tool for the outcome of the study evaluated by pain score, ROM, knee functional mobility using WOMAC, knee score, and functional score. The secondary measuring tool was patient’s satisfaction and duration for using gait aid. Continuous variables were expressed as mean ± standard deviation; median (minimum-maximum) is presented where appropriate. Categorical variables are presented as frequency and percentage. Difference of characteristics and all outcomes between two groups, the Student *t* test, or Mann-Whitney *U* test were used to compare continuous variables while the Chi-square test was compared for categorical variables. For all tests performed, a two-tailed *p* value < 0.05 was considered to be statistically significant. PASW Statistic (SPSS) 18.0 (SPSS, Inc., Chicago, IL, USA) was used to perform all statistical analyses.

## Results

Forty-eight patients were studied; 24 patients were the home visit program (TKR-H) and 24 were total knee replacement (TKR). The overall characteristics were males 14 (29.2%) and females 34 (70.8%). The average age was 64 years old and BMI was 25 kg/m^2^. The median pre-op pain score was 89 from 100 (range 89 to 90). The pre-op ROM (extension-flexion) was 0–100° in TKR group and 0–105° in TKR-H group. The comparison of sex, age, and BMI was not significantly different (*p* > 0.05) between TKR-H and TKR groups, while the median pre-op pain score was significantly lower than in TKR-H as compared with TKR (88 range 85–89 vs. 89 range 88–90, *p* = 0.007) (Table 1).

**Table 1:** General demographic was shown in table

Characteristics	Total	TKR-H	TKR	*P* value
	(*n* = 48)	(*n* = 24)	(*n* = 24)	
Sex (male), *n*(%)	14 (29.2%)	4 (16.7%)	10 (41.7%)	0.057
Age (years), mean ± SD	63.73 ± 5.52	63.63 ± 63	63.83 ± 4.93	0.898
BMI (kg/cm^2^), mean ± SD	25.26 ± 2.19	25.25 ± 2.35	25.28 ± 2.07	0.954
Pre-op pain score (0–100 min.), median (min-max)	88 (85–90)	88 (85–89)	89 (88–90)	0.007*
Pre-op ROM (extension-flexion)	–	0–105 (100%)	0–100 (100%)	NA

*P value < 0.05 was considered to be statistically significant

Whereas the level of pain score, ROM, gait aid independent, WOMAC, function scores, and knee score were statistically significant between TKR-H and TKR group. The pain score of TKR-H group was lower than TKR group (6.25 ± 10.13 and 35.67 ± 22.05; *p* <  0.001) and gait aid independent (2.75 ± 0.99 weeks and 3.71 ± 1.23 weeks; *p* = 0.005). The ROM of TKR-H group was higher than TKR group (107.71 ± 8.47 and 98.17 ± 9.57; *p* = 0.001), WOMAC (88.29 ± 10.66 and 68.00 ± 12.47; *p* <  0.001), function scores (77.83 ± 4.22 and 73.70 ± 7.48; *p* = 0.037), and knee score (81.67 ± 10.08 and 68.38 ± 6.45; *p* <  0.001). (Table 2).

**Table 2:** Data was shown pain score, ROM, gait aid independent, WOMAC, knee score, and function score respectively

Score	Total	TKR-H	TKR	*P* value
	(*n* = 48)	(*n* = 24)	(*n* = 24)	
Pain score, mean ± SD	20.96 ± 22.56	6.25 ± 10.13	35.67 ± 22.05	< 0.001*
ROM (°), mean ± SD	102.94 ± 10.15	107.71 ± 8.47	98.17 ± 9.57	0.001*
WOMAC, mean ± SD	79.58 ± 15.34	88.29 ± 10.66	68.00 ± 12.47	< 0.001*
Knee Score, mean ± SD	75.02 ± 10.73	81.67 ± 10.08	68.38 ± 6.45	< 0.001*
Function Score, mean ± SD	75.77 ± 6.36	77.83 ± 4.22	73.70 ± 7.48	0.037*
Patient’s satisfaction score	4.42 ± 1.31	4.71 ± 0.46	4.13 ± 0.45	< 0.001*
Gait aid independent (week), mean ± SD	3.23 ± 1.21	2.75 ± 0.99	3.71 ± 1.23	0.005*

*P value < 0.05 was considered to be statistically significant

**Fig. 2:**
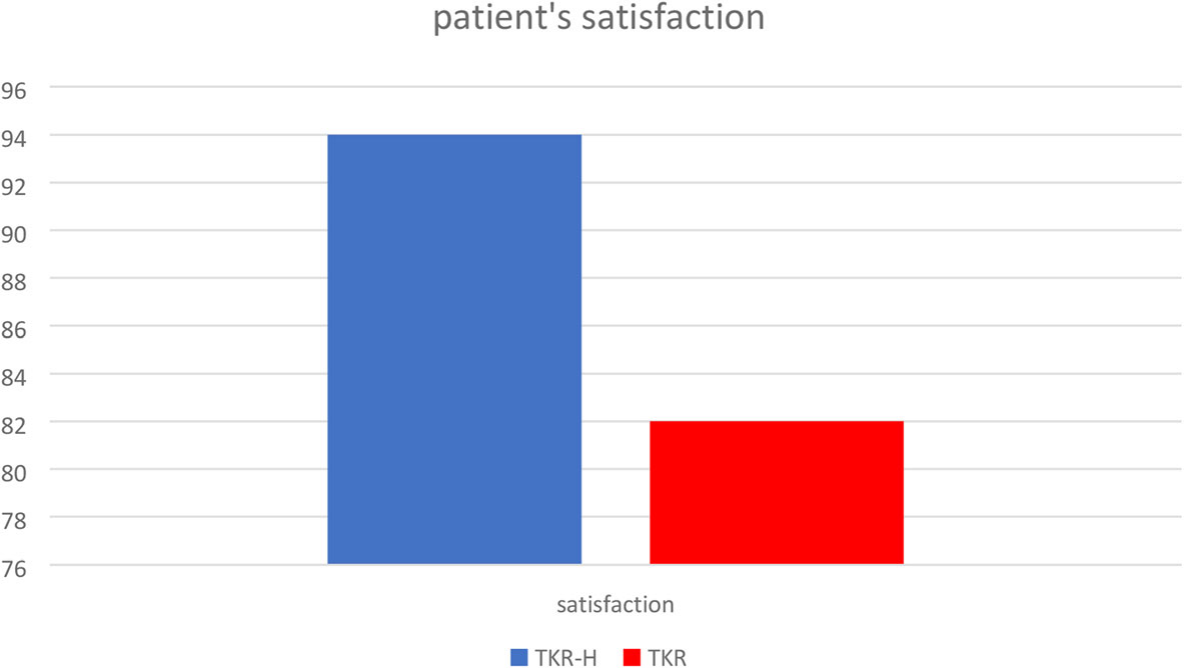
Summarized percentage of patient’s satisfaction between TKR-H and TKR

The patient satisfaction was 82% (average score was 4.13 ± 0.45 out of 5) for TKR group and 94% (average score was 4.71 ± 0.46 out of 5) for TKR-H group, and the satisfaction was significantly different between the two groups (*p* <  0.001) as shown in Table 2. We summarized the percentage of patient’s satisfaction between TKR-H and TKR group in Fig. 2. Both groups had no complications.

## Discussion

The patient satisfaction after TKR is associated with patient expectations, pain relief, and functional improvements. Lau et al. [17] suggested that two perspectives should be considered in the evaluation of patient satisfaction; internal determinants and external components. Internal determinants refer to patient-dependent factors, such as age, and expectations, whereas the external determinants indicate patient-independent factors, such as the hospital environment and the surgical techniques. Patient satisfaction after TKA has been described as ranging from 82 to 89%. There are many rating systems for assessing surgical outcome. The Patient Report Outcome Measurements (PROMs) in this study were related to WOMAC [18] and the Knee Society Score (KSS; 2011 version) [11]. The possible internal determinants of patient satisfaction include age, gender, patient personality, patient expectations, physical and psychological comorbidities, diagnosis for TKA, and the severity of the arthroplasty [20–23]. Whereas, the possible external components of patient satisfaction can be related to anesthesia, postoperative pain management, surgical technique, implant type, and postoperative rehabilitation. One study compared home-based rehabilitation with standard hospital rehabilitation in terms of improving knee joint mobility and recovery of muscle strength and function in patients after a total knee replacement. This study revealed that rehabilitation treatments offered either at home or in a hospital setting are equally effective. There have been no studies indicating that a home visit program improves patient satisfaction after a total knee replacement. Home visit programs might be classified as an external component based on the results of the study. It was found that for the pain score, the TKR-H is significantly better than TKR. The knee and function scores of TKR-H were significantly better than for TKR. The ROM and time to independent gait aid for TKR-H was found to be significantly better than for TKR. The patient satisfaction for TKR at 82% is the same as found in previous studies. The patient satisfaction for TKR-H was found to be 94% which is significantly higher than for TKR. Our mean WOMAC score is higher than the first 3 months. The mean WOMAC score was found to be the same for patients who did TKR at 1 year. Unique to this study is its rationale for home visits with activities related to PROMs. From home visits, we identified the patient’ s problems such as a poor environment, misunderstanding about the exercises, a lack of confidence in walking without a gait aid, misunderstanding about how to use a gait aid, a lack of wound care, and inappropriate nutrition. The home visit team, which consisted of the surgeon, nurses, physiotherapists, and nutritionists, advised and taught patients the correct and appropriate techniques and procedures. This helped strengthen their confidence in self-care, wound care, and nutrition while at home. The team also helped the families to empower the patients to have more confidence. Both the mindset of the patients and their families are very important. INHOMESSS provided the rationale for successful home visit activities (Additional file 1).

## Conclusion

We can have not only a good surgical outcome but also increased patient satisfaction with TKR with home visits. Our results showed that a home visit following TKR provides better results in every parameter than TKR without a home visit. The visit from the home visit team and good family care were found to be the key factors in improving satisfaction after a total knee replacement. Limitations of this study include the short period of time for TKR home visit, the cost, and the benefits which should be included in the next study.

## Supplementary information

**Additional file 1:** **Figure S1.** Condition of toilet. **Figure S2.** Nutrition advisor. **Figure S3.** Home environment. **Figure S4.** Other people. **Figure S5.** Medication. **Figure S6.** Physical examination.

## Data Availability

The Home Visit improved patient’s satisfaction after total knee replacement data used to support the findings of this study are included within this article.

## Abbreviations

BMI: Body mass index
INHOMESSS: Immobility, Nutrition, Home environment, Other people, Medications, Examination, Spiritual, Service Evaluation, Safety
PROMs: Patient report outcome measurements
ROM: Knee joint range of motion
TKR: Total knee replacement
TKR-H: Home visit program
VAS: Visual analog scale
WOMAC: The Western Ontario and McMaster Universities Arthritis Index
