# Supplementary material for: A home visit program versus a non-home visit program in total knee replacement patients: a randomized controlled trial
Source: J Orthop Surg Res. 2019 Nov 29;14:405. doi: 10.1186/s13018-019-1412-6 (PMC6884913; doi:10.1186/s13018-019-1412-6)
Supplement: Supplementary file 1 — Additional file 1: Figure S1. Condition of toilet. Figure S2. Nutrition advisor. Figure S3. Home environment. Figure S4. Other people. Figure S5. Medication. Figure S6. Physical examination. [file 13018_2019_1412_MOESM1_ESM.docx]

Additional file

In order to build relationships and trust for the patients, we used INHOMESSS as a success tool to meet the goal.

I = Immobility

We evaluated the patient's functional activities includes assessment of the activities of daily living (bathing, transfer, dressing, toileting, feeding, continence) and the instrumental activities of daily living (using the telephone, administering medications, paying bills, shopping for food, preparing meals, doing housework). We asked the patient to demonstrate elements of the daily routine, such as getting out of bed, performing personal hygiene and leisure activities, and getting in and out of a car. Corrective interventions can be directed at any deficiencies noted. For example, we suggested that the patient change the squat toilet to toilet seat and a hand rail to hold such as supplementary material figure-1. We accessed activities of daily living such as the way of shower, toilet, dressing, and instrumental activities of daily living.


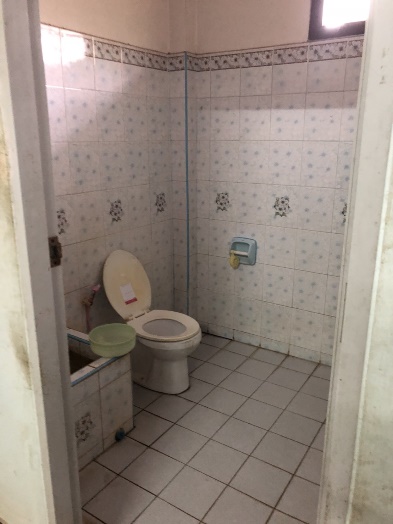

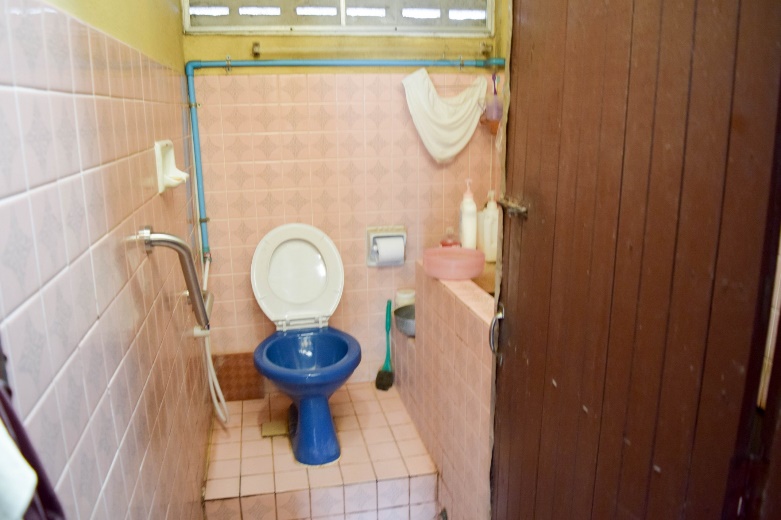


Figure S1. Condition of toilet

N = Nutrition

We assessed the patient's current state of nutrition, eating behaviors and food preferences. We always started by asking open-ended questions. For example, “We have been working hard on your diet to control your diabetes. Would you remember the types of foods you eat?” Improvements in material of cooking allow the physician to assess serving sizes and the nutritional value of foods with relative ease. How many meals do you have per one day? It is very surprise for this question. Because of the surgery we often found that patients always have meal more than 3 meals per one day because of the relatives came to visit at home and always bring food with them. We have nutritionist in the team and she always bring the food chart with her to show the healthy food as supplementary material figure-2. .


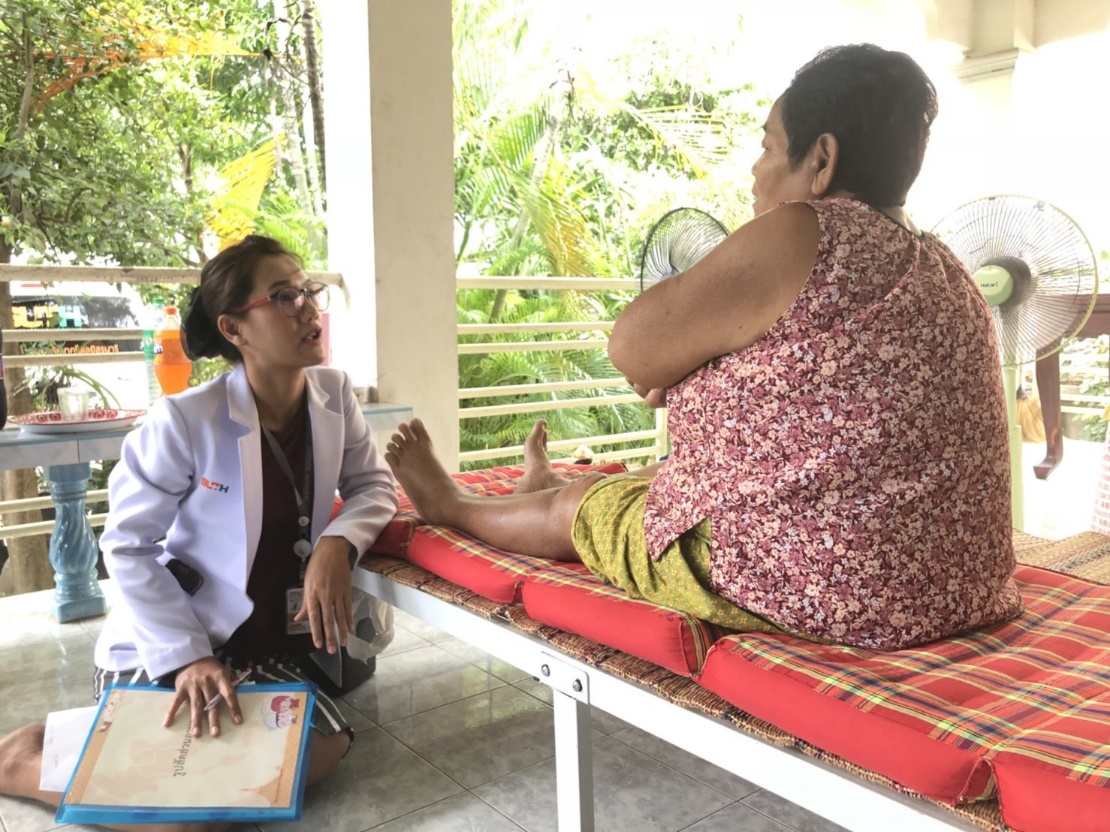


Figure S2. Nutrition advisor

H = Home Environment

The patient's home environment should allow for privacy, social interaction and both spiritual and emotional comfort and safety. A safe neighborhood with close proximity to services is important for many older patients. The home may reflect that patients and their families’ living condition, such as the presence of stairs in the house, the location of the bedroom downstairs or upstairs. As supplementary material figure-3.


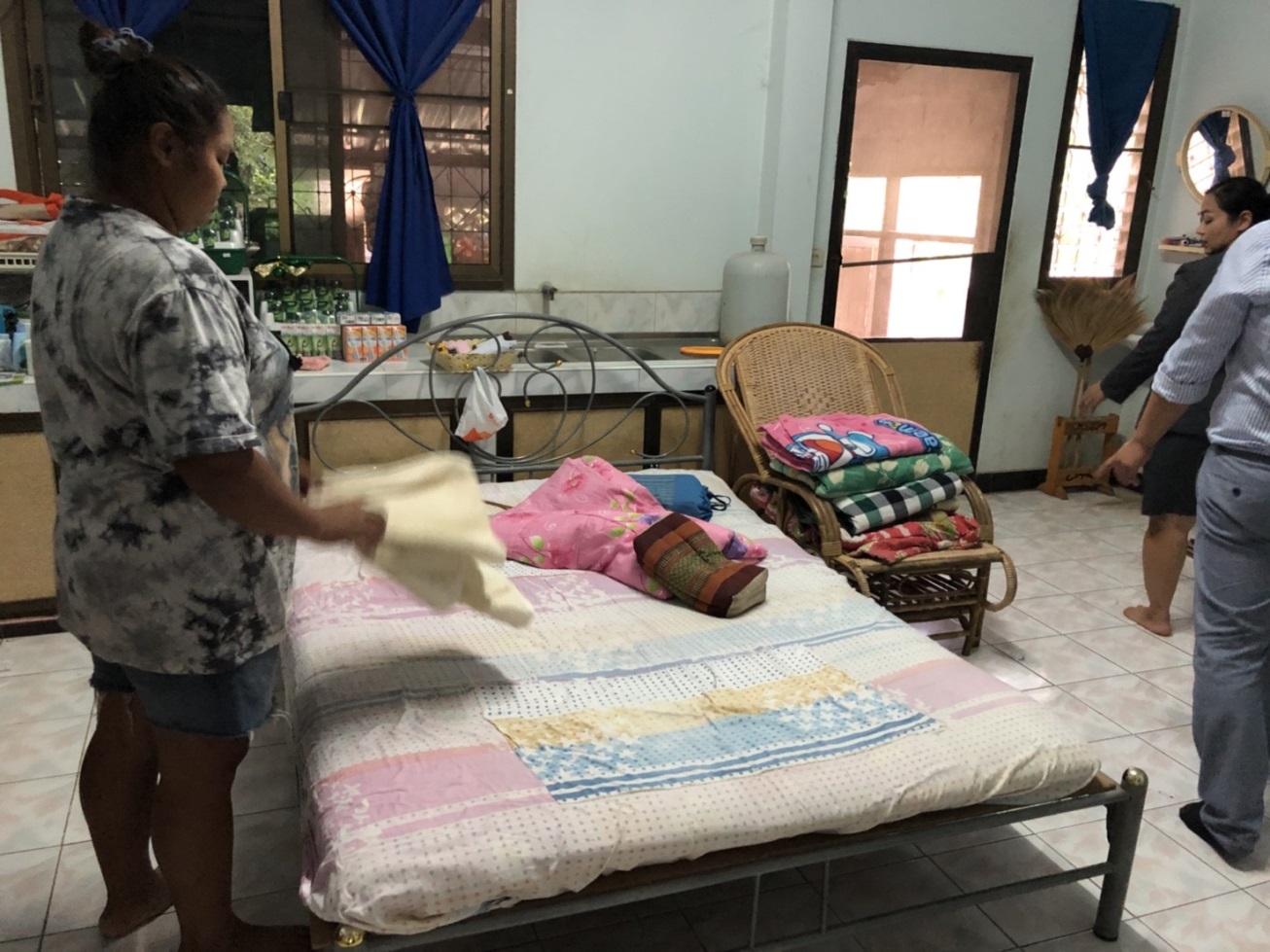


Figure S3. Home environment

O = Other people

Having the patient's social support system present at the home visit clarifies the roles and concerns of family members. We assessed the availability of emergency help for the patient from family members and friends and can clarify specific issues, such as who is to serve as surrogate for the patient in the event of incapacitation. Discussion of a living will be more comfortably performed during the home visit than in the usual clinic visit. Evaluation of the caregiver's needs and risk of burnout is critically important. As Supplementary material figure-4.


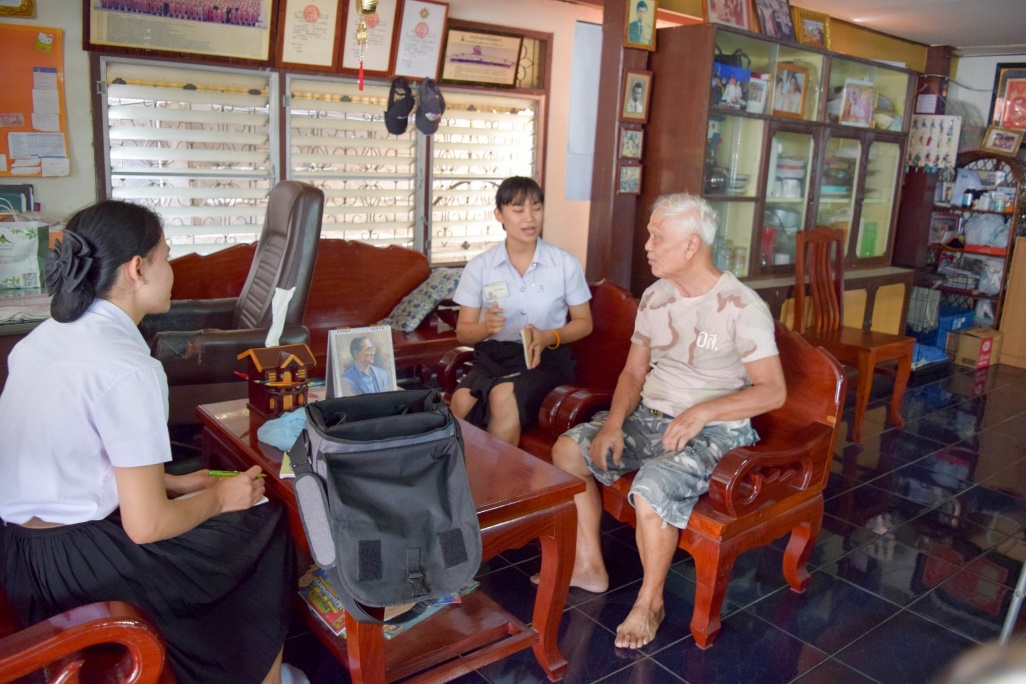


Figure S4. Other people

M = Medications

To remedy or avoid polypharmacy, we evaluated the type, amount and frequency of medications, and the organization and methods of medication delivery. An inventory of the patient's medicine container can provide clues to previously unidentified drug-drug or drug-food interactions. A home medication review can also allow a direct estimate of patient compliance. As supplementary material figure-5.


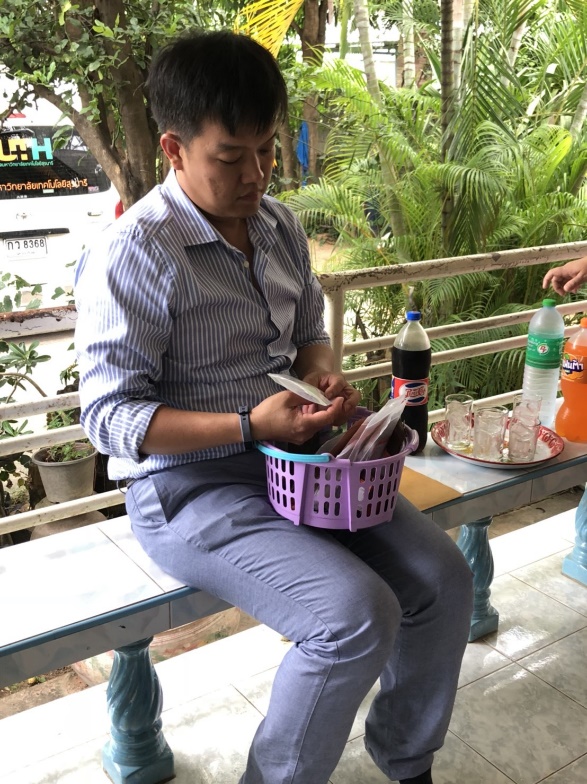


Figure S5. Medication

E = Examination

We do a directed physical examination based on the needs of the patient and the physician's agenda. We asked the patient demonstrate proper technique for the self-caring of walking with or without gait aid. In addition, we can weigh the patient and obtain a blood pressure measurement. In-person correlation of home and office measures provide useful information for future telephone and clinic contacts. As supplementary material figure-6.


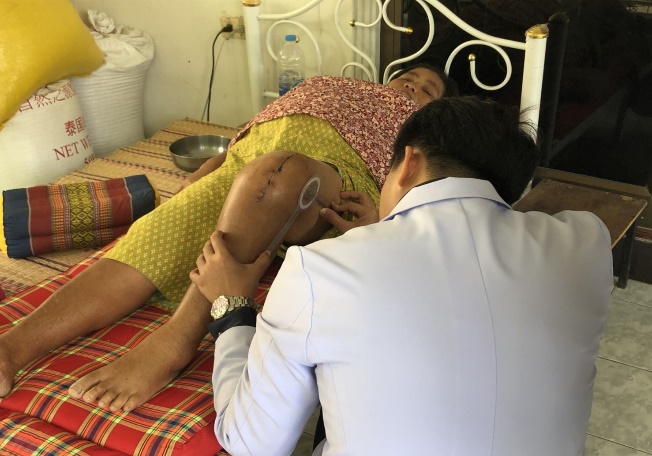

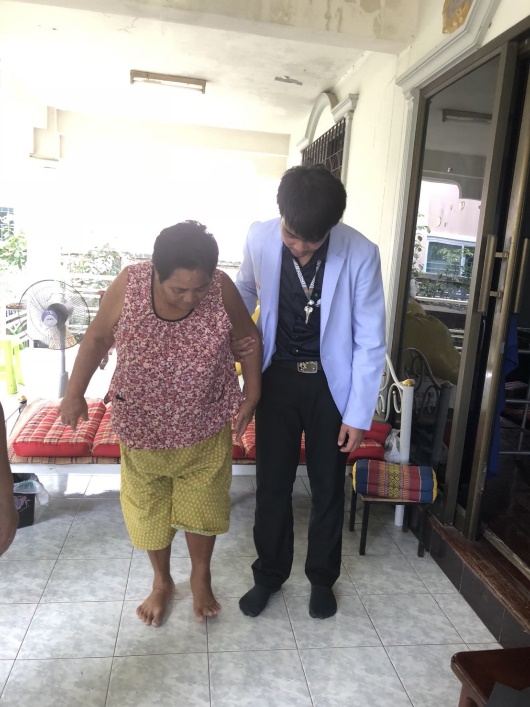


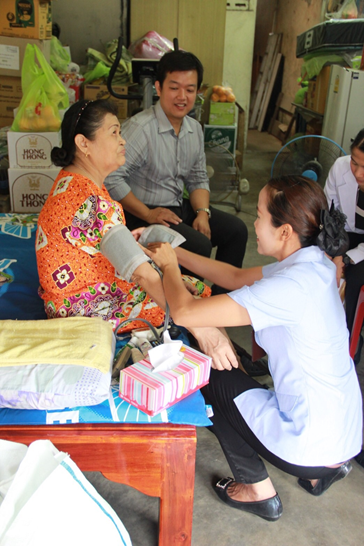


Figure S6. Physical examination

S = Safety

The goal of the home safety assessment is to determine whether the patient's environment is comfortable and safe (no unreasonable risk of injury). We identified and help modify potential safety hazards.

S = Spiritual Health

We asked about the influence of spiritual beliefs on the patient's sense of physical and emotional health. This information may provide the impetus, as desired by the patient, for a discussion of spirituality as a coping and healing strategy.

S = Services

We evaluated about the access health services for the patient and their family.
